# Plant responses to environmental stresses—from gene to biotechnology

**DOI:** 10.1093/aobpla/plx025

**Published:** 2017-06-27

**Authors:** Mohammad Abass Ahanger, Nudrat Aisha Akram, Muhammad Ashraf, Mohammed Nasser Alyemeni, Leonard Wijaya, Parvaiz Ahmad

**Affiliations:** 1 School of Studies in Botany, Jiwaji University, Gwalior, MP 474011, India; 2 Department of Botany, Government College University, Faisalabad 38000, Pakistan; 3 Pakistan Science Foundation, Islamabad, Pakistan; 4 Department of Botany & Microbiology, King Saud University, Riyadh, Saudi Arabia; 5 Department of Botany, S.P. College, Srinagar, Jammu and Kashmir 190001, India

**Keywords:** Abiotic stresses, biotechnology, cold tolerance, ion transporters, pathogens, stress tolerance, transgenics

## Abstract

Increasing global population, urbanization and industrialization are increasing the rate of conversion of arable land into wasteland. Supplying food to an ever-increasing population is one of the biggest challenges that agriculturalists and plant scientists are currently confronting. Environmental stresses make this situation even graver. Despite the induction of several tolerance mechanisms, sensitive plants often fail to survive under environmental extremes. New technological approaches are imperative. Conventional breeding methods have a limited potential to improve plant genomes against environmental stress. Recently, genetic engineering has contributed enormously to the development of genetically modified varieties of different crops such as cotton, maize, rice, canola and soybean. The identification of stress-responsive genes and their subsequent introgression or overexpression within sensitive crop species are now being widely carried out by plant scientists. Engineering of important tolerance pathways, like antioxidant enzymes, osmolyte accumulation, membrane-localized transporters for efficient compartmentation of deleterious ions and accumulation of essential elements and resistance against pests or pathogens is also an area that has been intensively researched. In this review, the role of biotechnology and its successes, prospects and challenges in developing stress-tolerant crop cultivars are discussed.

## Teaser

Supplying food to an ever-increasing population is one of the biggest challenges worldwide. Environmental stresses make this situation even graver. New technological approaches are imperative. Recently, genetic engineering has contributed enormously to the development of genetically modified varieties of different crops such as cotton, maize, rice, canola and soybean. Identification of stress-responsive genes and their subsequent introgression or overexpression within sensitive crop species are now being widely carried out by plant scientists. In this review, the role of biotechnology in association with genomics and its successes, endeavours, prospects and challenges in developing stress-tolerant crop cultivars are discussed.

## Introduction

In plant biology, the transgenic approach has emerged as an important tool to adapt crops to rapidly changing environmental conditions. The use of transgenic crops has increased considerably over the past decade. The primary step before proceeding with transgenics is the identification of genes serving as key regulators of different metabolic pathways, including osmolyte synthesis, ion homeostasis through selective ion uptake, antioxidant defence system and other frontline defence pathways (Ahmad *et al.* 2012).

Genome editing has revolutionized plant biotechnology by providing plant scientists with the option of selection or incorporation of genes of interest into desired species or cultivars (Tzfira *et al.* 2012). A particular stress alters the expression of specific genes in a species-dependent fashion. It causes differences in the efficiency of signal perception and subsequent transcriptional alterations leading to elicitation of a specific response and adaptation and finally enhanced stress tolerance. The microarray hybridization technique (employing cDNAs or oligonucleotides) is the main technique used to isolate a set of desired genes. Recently, rice microarrays using oligonucleotides (22 000 oligoarray) have been produced based on full-length cDNA information through the Rice Genome Program of Japan. A number of companies such as Axon Instruments, Inc., Amersham Biosciences, MWG Biotech AG, Genetic Analysis Technology Consortium, Clontech Laboratories, Azign Bioscience A/S, Mergen Ltd., Invitrogen, Promega, QIAGEN, Stratagene and QIAGEN Operon are providing these arrays. The services provided by Agilent Company Ltd. are now being used to evaluate and understand responses to abiotic stresses in rice through transcriptomic studies (Ban and Moriguchi 2010). Limitations in terms of the availability of sophisticated cost-intensive apparatus and materials are common in many countries’ laboratories (Ban and Moriguchi 2010).

Suppression Subtraction Hybridization (SSH) is an extremely powerful and widely used technique for separating cDNA or genomic DNA (Ban and Moriguchi 2010; Ding *et al.* 2014; Ma *et al.* 2017). Transgenes are introduced into plants by biological or physical methods. Successful and efficient transformation demands specific criteria to be met including regeneration capacity and competence of target tissues, efficient DNA delivery method and precautions for avoiding somaclonal variations and sterility. Several techniques fulfil these requirements, e.g. protoplast transformation, biolistics or microprojectile bombardment and *Agrobacterium*-mediated transformation (Rodrigues *et al.* 2012; Fei *et al.* 2015).

In this review, we summarize stress-responsive genes and their subsequent introgression or overexpression within other crop species. In addition, engineering of important pathways involved in the oxidative defence system, osmoprotection, ion transportation and resistance against pathogens is explored. The role of biotechnology and its successes, prospects and challenges in developing stress-tolerant crop cultivars are discussed.

## Responses of Transgenic Plants to Different Stresses

The past decade has extensively increased our understanding of ways to improve stress tolerance through the transgenic approach (Bhatnagar-Mathur *et al.* 2008; Gilliham *et al.* 2016; Wang *et al.* 2016). The majority of transgenic plants has been tested against different abiotic factors only in growth chambers, greenhouses or under controlled conditions (Ashraf and Foolad 2007). Few studies are available in the literature in which abiotic stress tolerant transgenic plants were tested under true field conditions (Table 1).

**Table 1.** Transgenic plants showing resistance to various environmental stresses through expression of genes.

Plant/crop species	Gene	Possible role	Tolerance to	Growth conditions	Reference
*Arabidopsis thaliana*	Mitochondrial penta tricopeptide repeat domain protein (PPR40)	Increase in proline, mitochondrial respiration. Decrease in SOD, APX, lipid peroxidation	Salinity stress	Growth chamber	Zsigmond *et al.* 2012
*Arabidopsis thaliana*	*Ataox1*	Decreased ROS, decreased Na^+^ and increased potassium in shoot, increased AOX activity	Salinity stress	Glasshouse	Smith *et al.* 2009
*Arabidopsis thaliana*	P5CS genes from common bean	Higher proline content	Salinity stress	Greenhouse	Chen *et al.* 2013
Arabidopsis and tobacco	β-lycopene cyclase gene SeLCY from *Salicornia europaea*	Efficient scavenging of ROS by enhanced carotenoid contents	Salinity stress	Greenhouse	Chen *et al.* 2011
Tobacco	Dehydration-responsive RD22 gene of *Vitis vinifera*	Increase in germination, chlorophyll and osmotic constituents like sugars with a concomitant decrease in Na uptake	Salinity stress	Greenhouse	Jamoussi *et al.* 2014
*Arabidopsis thaliana*	Zeaxanthin epoxidase (ZEP)	ABA biosynthesis and xanthophyll cycle	Salinity, drought	Controlled conditions	Park *et al.* 2008
*Brassica juncea*	γ-Tocopherol methyl transferase (γ-TMT)	Six-fold increase in γ-tocopherol	Salinity, osmotic and heavy metal stress	Hydroponics under controlled conditions	Yusuf *et al.* 2010
Tomato	Osmotin	Increased proline, RWC, germination	Salinity and drought	Controlled conditions	Goel *et al.* 2010
*Arabidopsis thaliana*	GmWRKY13, GmWRKY21 and GmWRKY54	Lateral root development	Drought, salinity and cold	Controlled conditions	Zhou *et al.* 2008
Tomato	CaXTH3, a hot pepper xyloglucan endotransglucosylase/hydrolase	Maintained sufficient chlorophyll even at 100 mM NaCl	Drought and salinity	Controlled conditions	Choi *et al.* 2011
Sweet potato (*Ipomoea batatas*)	Chloroplastic BADH gene from *Spinacia oleracea* (SoBADH)	Glycine betaine accumulation, maintained cell membrane integrity, photosynthetic activity.	Salinity, oxidative and low temperature stress	Growth chamber	Fan *et al.* 2012
		Reduced ROS production and quick ROS scavenging by increased activity of free radical-scavenging enzymes			
Maize	Rab28 LEA	Increase RWC, leaf and root growth, lower MDA	Water stress	Greenhouse	Amara *et al.* 2013
*Nicotiana tabacum* cv. Xanthi)	Cu/Zn sod (cytsod) from *Spinacia oleracea* and cytosolic apx1 (cytapx) from *Pisum sativum*	Higher WUE, photosynthetic rates, SOD and APX activity. Reduced lipid peroxidation, H_2_O_2_	Water stress	Greenhouse	Faize *et al.* 2011
Tobacco	PtrABF, a bZIP transcription factor	Decreased ROS	Drought	Growth chamber	Huang *et al.* 2010
		Increased activities of SOD, CAT and CAT			
		Overexpression of stress responsive genes			
*Arabidopsis thaliana*	TaPLDα	High RWC, less membrane leakage and chlorosis	Drought and osmotic stress	Growth chamber	Wang *et al.* 2014
*Arabidopsis thaliana*	*Medicago sativa* (alfalfa) helicase 1(MH1), homolog of the pea DNA helicases 45 (PDH45)	Increased SOD and APX activities and proline content; osmotic adjustment	Mannitol, NaCl, methyl viologen and ABA	Growth chamber	Luo *et al.* 2009
Tobacco	*MtSAP1*	Overexpression of MtSAP1 enhanced stress tolerance	Temperature stress, drastic osmotic and salinity stress	Controlled conditions	Charrier *et al.* 2013
Tobacco	S-adenosylmethionine decarboxylase	Increased polyamine biosynthesis	Osmotic stress, oxidative stress, temperature stress, acid stress	Room conditions	Wi *et al.* 2006
	Gene (SAMDC) from *Dianthus caryophyllus*				
Tobacco	Arabidopsis phytochelatin synthase gene (AtPCS1)	Heavy metal detoxification	Cadmium stress	Greenhouse hydroponics	Pomponi *et al.* 2006
Arabidopsis and tomato	Tryptophansynthase beta1 (AtTSB1)	High chlorophyll and tryptophan	Cadmium stress	Controlled conditions	Sanjaya *et al.* 2008
		Synthase β enzyme activity, low lipid peroxidation			
Fescue plants	2-Cys peroxiredoxins (2-Cys Prx)	Reduced lipid peroxidation and electrolyte leakage, Maintained chlorophyll fluorescence	Methyleviologen, heat stress	Growth chamber	Kim *et al.* 2010
Indica rice	Cytosolic copper/zinc superoxide dismutase (CuZnSOD) from *Avicennia marina*	Higher activity of SOD	Methylviologen, oxidative stress, salinity stress	Growth chamber	Prashanth *et al.* 2008
*Solanum tuberosum* L.	Nucleoside diphosphate kinase 2 (AtNDPK2)	Antioxidant gene regulation and high antioxidant enzyme activity	Methyl viologen (MV), oxidative stress, high temperature and salt stress	Growth chamber	Tang *et al.* 2008
Tobacco	Wheat OXO (oxalate oxidase) gene	Higher activities SOD, CAT, APX and GR. Increased photosynthetic efficiency.	Methyl viologen (MV) and high temperature oxidative stress	Greenhouse	Wan *et al.* 2009
*Arabidopsis thaliana*	*OsSPX1*	Increased proline and decreased MDA	Cold stress	Glasshouse	Zhao *et al.* 2009
*Arabidopsis thaliana*	OsHsfA2e TF from rice	Increased expression of stress associated genes	High temperature	Controlled conditions	Yokotani *et al.* 2008
Tobacco	NPR1 (non-expressor of pathogenesis related genes 1, AtNPR1	Modulates salicylic acid mediated system acquired resistance; Cross-talk with jasmonate pathway	Oxidative stress	Culture room	Srinivasan *et al.* 2009
*Arabidopsis thaliana*	MSD1, CAT1, HPT1	High activities of SOD and CAT, and increased tocopherol content	Oxidative stress	Growth chamber	Xi *et al.* 2010
*Arabidopsis thaliana*	codA gene from *Arthrobacter globiformis*	High glycine betaine synthesis, maintained photosynthesis through protection to PSII; higher activity of APX and CAT	Light stress	Controlled conditions	Alia *et al.* 1999

### Drought stress

Adaptation to water stress conditions is one of the major challenges for plant scientists and biotechnologists in the current scenario of rapid climate change. Scientists are increasing their efforts to elucidate various climate triggered metabolic processes at cellular and gene levels (Chaves *et al.* 2003). Research trials tailoring the plant genome for water stress tolerance and enhanced yield carried out all over the globe have increased with the premier goal of more crop per drop (Medici *et al.* 2014). There is a growing trend to improve the water use efficiency (WUE) of crops to enable the more efficient use of available water (Al-Karaki 2000).

The difference between transgenic and conventional approaches for achieving improved water stress tolerance is considerable. One viable transgenic approach is the engineering of genes of important metabolic and defensive pathways, e.g. osmoprotectant synthesizing pathways and antioxidant defence systems (Ashraf 2009; Wang *et al.* 2016). Several stress inducible genes have been identified through microarrays, but as yet their function within the molecular mechanisms for crop stress response and tolerance still needs to be deciphered. For example, the production of the phytohormone abscisic acid (ABA) causes stomatal closure and induces the expression of stress responsive genes (Tuteja 2007). However, how they function is not known. In *Arabidopsis thaliana* wild type and abi11 mutant seedlings, Hoth *et al.* (2002) identified ∼1354 genes that were up- or down-regulated following ABA treatment, with most of them coding for signal transduction.

Pinheiro and Chaves (2011) state that during lowered stomatal conductance, in combination with sustained irradiance, relatively more CO_2_ is available within intercellular sites, because during the Calvin cycle the consumption of light is slowed down while production rates of reducing power are increased under such conditions. These changes slow down the photosynthetic rate through photoinhibition, which may serve as a defensive mechanism for plants following the C_3_ pathway through thermo-regulated energy dissipation and light harvesting complexes (Ruban *et al.* 2012). Plants with upregulated photosynthetic pathways exhibit a high rate of photosynthesis (Gu *et al.* 2013).

There are three major pathways of CO_2_ assimilation and fixation: C_3_, C_4_ and crassulacean acid metabolism (CAM) pathways. C_4_ plants can minimize photorespiration by separating initial CO_2_ fixation and the Calvin cycle in different cell types, and CAM plants can fix carbon at night, and are more tolerant to drought stress due to their more efficient carbon fixation and specialized anatomical features (Ashraf and Harris 2013). Recently, Ashraf and Harris (2013) comprehensively described the progress made during the last two decades in producing transgenic lines of different C_3_ crops with enhanced photosynthetic performance either due to introgression of genes encoding C_4_ enzymes into C_3_ plants or overexpression of C_3_ enzymes or transcription factors (TF). Usually, C_4_ and CAM plants are best adapted to arid environments, because they have higher photosynthetic efficiency as well as WUE as compared with C_3_ plants (Fischer and Turner 1978). Likewise, Kim *et al.* (2014) reported that overexpression of *Capsicum annuum* drought stress responsive 6 (CaDSR6) in *Arabidopsis* plants led to higher tolerance to drought as compared with wild type plants. Saad *et al.* (2013) also showed that the stress-responsive NAC1 (*SNAC1*) gene controlled signalling of sucrose phosphate synthase type 2C protein phosphatases, 1-phosphatidylinositol-3-phosphate-5-kinase as well as regulatory components of ABA receptor in wheat plants under drought stress. Overall, a variety of genes contributing to drought tolerance in plants have been explored and characterized in *Arabidopsis*. However, few of these genes have been tested in other crops, and only under controlled or laboratory conditions instead of natural field conditions.

### Salinity stress

Salt is a premier environmental stress that affects plant growth and development adversely through induction of ion toxicity, reduced water uptake, hormonal disturbance and oxidative stress (Ashraf and McNeilly 2004; Athar *et al.* 2008; Tuna *et al.* 2007; Siddiqi *et al.* 2007; Ashraf and Foolad 2013). As with other abiotic stresses, several tolerance responses are triggered in the plants to avoid high salinity-induced deleterious effects. One response used to avoid saline stress is compartmentation and the exclusion of deleterious ions (Na^+^ and Cl^-^) from sensitive tissues like the mesophyll (where sodium toxicity is induced by competing for K^+^ binding sites) and their diversion into the apoplast or vacuole (Sperling *et al.* 2014).

Maintenance of high potassium and retention of deleterious ions or solutes within a root or apoplastic regions are the major tolerance strategies, and hence a high K/Na ratio is maintained through the efficient function of transporters (Shabala and Cuin 2008). Pandolfi *et al.* (2012) suggested that short-term exposure and acclimation of glycophytes to a lower salt concentration can help withstand prolonged exposure to a higher concentration. The plant acclimates through a set of physiological mechanisms including controlled xylem ion loading and efficient Na^+^ compartmentation (Pandolfi *et al.* 2012).

For example, plants overexpressing the ion transporter genes show high salinity tolerance as in halophytes (Flowers and Colmer 2008). Overexpression of Na^+^/H^+^ antiporter (AlNHX) from the halophyte, *Aeluropuslittoralis*, in tobacco enhanced the salinity tolerance of tobacco by maintaining a suitable level of Na^+^ and K^+^/Na^+^ ratio (Zhang *et al.* 2008). Overexpression of vacuolar ATPase subunit c1 (*SaVHAc1*) gene from *Spartinaalterniflora* enhanced rates of photosynthesis and cell wall expansion, improved the K^+^/Na^+^ ratio and led to a higher relative water content (RWC) in rice (Baisakh *et al.* 2012). Overexpression of the wheat transporter gene*TaNHX2* enhanced the salt tolerance of *C.annuum* by improving the K^+^/Na^+^ ratio (Bulle *et al.* 2016). These studies indicate that genes contributing towards tolerance to high salinity in halophytic grasses could be better engineered to achieve enhanced tolerance of sensitive cash crops.

### Cold stress

Plant survival under low temperature depends on the physiological and molecular responses triggered by the plant on exposure to low temperature (Sergeant *et al.* 2014; John *et al.* 2016). These can be confounded by photoperiod response as cold is often associated with extreme latitudes. Water availability, growth and development, energy metabolism and photoperiod are amongst the important factors that determine the deacclimation and reacclimation of plants to cold stress (Thomashow 1999). Compatible solutes, membrane proteins, antioxidants and expression of cold responsive genes have a significant role in cold tolerance (Kalberer *et al.* 2006). Cold stress alters the expression of putative cold responsive genes coding for an array of important proteins, for example enzymes involved in respiration and the metabolism of carbohydrates, phenylpropanoids, lipids, antioxidants and those coding for chaperones and antifreeze proteins. Several other genes involved in regulating intriguing tolerance mechanisms are involved in freezing-induced dehydration (John *et al.* 2016). Altered gene expression and subsequent production of specific proteins during cold tolerance play an important role in the distribution and survival of plants as well as yield (Sanghera *et al.* 2011).

Interspecific and intergeneric hybridization-dependent conventional breeding has not been fully successful in developing cold tolerant crop cultivars. However, biotechnological and molecular approaches, including genome sequencing and alteration of the genome for transgenic development, provide an opportunity to understand and access the complex cold tolerance mechanisms operating at the transcriptional as well as the translational levels (John *et al.* 2016). Altered gene expression has increased the level of several metabolites that have a protective role under cold stress. Amongst the low-temperature-induced genes that have been isolated to date, the expression of most of them has been reported to be regulated by cold binding factor/dehydration responsive element binding transcription factors (CBF/DREB TFs; Sanghera *et al.* 2011).

Cold stress induces the expression of several proteins, e.g. proteins of methionine pathways and membrane stabilizing proteins. The methionine metabolism pathway has an important role in the biosynthesis of essential metabolites including polyols and polyamines, which play a role in cold acclimation. Although their actual role in cold tolerance is not fully known, their accumulation in plants has been reported in response to cold stress (John *et al.* 2016). Overexpression of methionine sulphoxide reductase A (MsrA), an important enzyme in the regulation of methionine metabolism, increases resistance to oxidative damage at low temperatures. For example, *Arabidopsis* plants with a mutation in methionine sulphoxide reductase B3 (MsrB3) were more sensitive to low temperature than their respective wild-type and MsrB3 transgenic plants. MsrB3 plays a ubiquitous role in eliminating reactive oxygen species (ROS) and methionine sulphoxide (MetO) accumulating in the endoplasmic reticulum during cold stress (Kwon *et al.* 2007).

Cold stress is believed to damage photosynthetic machinery, including photosystems and photosynthetic pigments, by altering the expression of photosynthetic genes (Oquist and Huner 2003). Han *et al.* (2010) isolated the violaxanthin de-epoxidase gene (*LeVDE*), a gene regulated by temperature rhythms, from *Lycopersicon esculentum*. Overexpression of this gene increased non-photochemical quenching, *F_v_*/*F_m_*and quantum yield, oxidizable P700, and the activity of the xanthophyll cycle and alleviated PSI and PSII photoinhibition under temperature stress. Recently, similar observations have been reported in transgenic tobacco by introgression of *LeLUT1* (carotenoid epsilon-ring hydroxylase gene from tomato), which reduced ROS production and hence maintained membrane integrity (Miller *et al.* 2010). Transgenic plants that overexpress these stress responsive genes benefit from their key roles in alleviating photoinhibition and photo-oxidation, which in turn decrease the sensitivity of the plant’s photosynthetic apparatus to cold (Zhou *et al.* 2013). The gene *AtICE1*, which is responsible for stimulating the expression of CBF/DREB in *Arabidopsis* under cold stress, was introgressed in rice thereby enhancing tolerance to cold stress (Dian-jun *et al.* 2008). Transgenic *A. thaliana* overexpressing *CcCDR*, a potent cold and drought regulatory protein gene, conferred enhanced tolerance to cold, salinity and low temperature by improving various physio-biochemical attributes, such as increased antioxidant activity and accumulation of osmolytes (Tamirisa *et al.* 2014).

By using suppression subtractive hybridization (SSH), Guo *et al.* (2013) identified the genes up- or down-regulated in ABA-pre-treated pepper seedlings incubated at 6 °C for two days. It has been observed that 50.68 % of unigenes showed similarities to genes with known functions while 49.32 % showed fewer similarities or unknown functions. The expression level of ten genes was at least 2-fold higher in the ABA-pre-treated seedlings than in non-treated (control) plants under chilling stress, which suggested that ABA negatively or positively regulates the genes in pepper plants under cold stress.

Cold induces accumulation of oligosaccharides and galactosyl synthase activity. Galactinol synthase mediates the synthesis of galactinol, which serves as a donor of galactosyl during the synthesis of oligosaccharides of the raffinose family (Zhou *et al.* 2013). *Photinia serrulata* overexpressing the galactinol synthase gene (*AmGSl*) from a cold tolerant tree, *Ammopiptanthus mongolicus*, exhibited enhanced cold tolerance (Song *et al.* 2013). Galactinol and raffinose are active scavengers of hydroxyl radicals. The role of galactinol synthase in drought and salinity is well documented (Nishizawa *et al.* 2008), yet very few reports are available pertaining to its possible role in cold tolerance. One of the few studies is by Zhou *et al.* (2013), who introgressed and overexpressed *MfGolS1* in tobacco, which resulted in increased cold tolerance through improved formation of galactinol, stachyose and raffinose. Elucidation of mechanisms of tolerance to cold stress in cold tolerant grasses at biochemical and molecular levels can be very helpful in improving our understanding of putative cold responsive genes and their subsequent introgression for enhancing the tolerance of economic crops to cold stress.

### High temperature

High temperature reduces a number of growth and physiological processes including seed germination, subsequent development, reproductive processes and photosynthesis, which have adverse effects on the overall yield of a crop (Gillooly *et al.* 2001). For example, impaired reproductive growth by high temperature results in inhibition of pollen grain swelling leading to anther indehiscence and perturbed pollen dispersal, which ultimately adversely affects seed production (Das *et al.* 2014). Understanding the high temperature tolerance mechanisms at physiological, biochemical and molecular levels in the light of global warming is essential for further successful efforts in developing high temperature tolerant crop cultivars. Genetic and molecular mechanisms for circumventing high-temperature-induced deleterious changes play an essential role in plant survival under such conditions. Sensing of high temperature stress and developing tolerance is highly complex, involving networks operating in different cellular compartments. Different putative sensors, e.g. histone sensors located in the nucleus, protein sensors in the endoplasmic reticulum and cytoplasm and a plasma membrane channel initiating inward calcium flux, mediate activation of heat stress responsive genes involved in thermotolerance (Mittler *et al.* 2012).

Genome modification for thermotolerance in crop plants is of immense concern because of its direct influence on the mechanisms involved in the reprogramming of the proteome, transcriptome, metabolome and lipidome. Molecular chaperones, e.g. heat shock proteins (HSPs), have a key role in mitigating the deleterious effects induced by heat stress (Xu *et al.* 2013). Reduction in the levels of HSPs causes developmental abnormalities (Kotak *et al.* 2007). Five major highly conserved HSP families have been recognized that differ in their respective molecular masses. Under normal metabolism, the HSPs are involved in several processes including protein folding, assembly, translocation as well as degradation, signalling and cell cycle control (Young *et al.* 2001). However, under stress conditions, the HSPs interact with other co-chaperones to bring about refolding of proteins in order to re-establish protein conformation and cellular homoeostasis, thereby protecting plant cellular functioning.

The essence behind the successful acclimation of plants to high temperature depends on the massive accumulation of transcripts coding for HSPs and ROS detoxifying enzymes like ascorbate peroxidase (APX). For example, *Zea mays* and *Arabidopsis* mutants for HSP100 showed retarded growth and adaptation to high temperature (Hong and Vierling 2000; Nieto-Sotelo *et al.* 2002). Similarly, silencing of chloroplast HSP100/ClpB protein gene expression in tomato reduced heat stress tolerance (Yang *et al.* 2006). Reports pertaining to the sensitivity of crop plants to high temperature as a result of mutation/silencing of HSPs (Bita and Gerats 2013) help our understanding of how essential these HSPs are for plants in triggering expression of heat responsive genes. Thermotolerance in plants can be better achieved by manipulating the detoxification pathways of ROS, e.g. Shi *et al.* (2001) cloned the peroxisomal APX-encoding gene, *HvAPX1*, and its introgression within *Arabidopsis* enhanced heat stress tolerance by increasing APX activity, thereby exhibiting low lipid peroxidation. The phospholipid hydroperoxide glutathione peroxidase encoding gene from *L.esculentum*, *LePHGPx*, protects yeast cells from lethal effects. However, its introgression and overexpression protected tomato from lethal temperature and salinity levels by reducing apoptosis levels (Chen *et al.* 2004).

Until recently, the genes that have been identified or introgressed in different genetically modified (GM) plants mainly relate to the regulation of the oxidative defence system. However, multiple other plant metabolic systems and activities are affected by changes in temperature, and have the potential to be tackled in transgenic crops. Moreover, a rise in ambient temperature as already visible over the last 10 years is a continuing challenge for crop productivity, creating the need to develop stress tolerant plants with heat tolerance.

### Fungicide and herbicide stress resistance

A variety of pesticides, herbicides and fungicides are frequently used to control crop loss due to pathogen attack (Yoon *et al.* 2013). Excessive use of these chemicals has a considerable negative impact on crop growth and yield (Chen 2006). Use of pesticides, fungicides and herbicides has become an integral part of modern agriculture (Aktar *et al.* 2009; Mattah *et al.* 2015). Residues of sprayed pesticides and fungicides residing on the fruits or seeds have a direct impact on human health. Scientists are continuously endeavouring to develop alternative chemicals to replace the commonly used chemicals so that threats to plants, animals as well as the environment can be minimized (Aktar *et al.* 2009; Mahmood *et al.* 2014; Mattah *et al.* 2015). Crops vary in their degree of sensitivity towards a particular pesticide, herbicide or fungicide, and extreme conditions in terms of heavy use of these chemicals can lead to crop death because of their direct interference with the metabolic processes of the plant (Mahmood *et al.* 2014; http://www.irac-online.org).

Synthetic pesticides, herbicides and fungicides are effective, but excessive use can generate environmental pollution, development of resistance and non-degradable residues. For example, chemical fungicides used for the treatment of plant diseases have diverse mechanisms of action involving the mitochondrial respiratory chain, inhibition of sterol biosynthesis as well as microtubule assembly, resulting in some limitations related to their toxicity and resistance in plants. A number of stress response pathways such as the cell wall integrity and high-osmolarity glycerol pathway are triggered by stimuli such as changes in osmolarity, cell wall instability and production of ROS (Hayes *et al.* 2014). There is, however, an emerging fear globally about the mis/over use of synthetic chemicals particularly on food crops because of their potential effects on the environment and human health (Al-Samarrai *et al.* 2012; Yoon *et al.* 2013). So, the introduction of bio pesticides/fungicides/herbicides is necessary (Yoon *et al.* 2013).

Elucidating and understanding the molecular mechanisms of chemicals used to control pests, and the development of pest-resistant crops and other alternative ecologically sound methods, are important so that chemical-dependent agriculture can be replaced with safer productive alternatives to agrochemicals. The development of crops that are resistant to pests and fungi and improving plant tolerance to a particular chemical pesticide or fungicide are being discussed in this regard (Mahmood *et al.* 2014). In addition to causing crop damage, many insects and pests have developed resistance to these chemicals, e.g. in pests, resistance mediated through enhanced activities of complex multigene enzymes like glutathione-*S*-transferase, esterases and cytochrome P450s is well reported in the literature (Bass and Field 2011). Engineering of crop plants by introducing genes involved in these important defence mechanisms from animals, bacteria and pests could be a useful part of xenobiotic strategies (Abhilash *et al.* 2009).

Qian*et al.* (2014) demonstrated that introgression of α-momorcharin (α-MC), a ribosome-inactivating protein (RIP) isolated from *Momordicacharantia* seeds, enhanced the tolerance of rice to *Magnaporthe grisea* induced blast. In another study, Zhu *et al.* (2013) showed that pre-treatment of tobacco plants with α-MC (0.5 mg mL^-1^) increased resistance to *Bipolaris maydis*, *Fusarium graminearum*, *Aspergillus oryzae*, *Aspergillus niger* and *Sclerotinia sclerotiorum*, thereby favouring the antifungal and antiviral activity of α-MC.

Use of fungal cell wall degrading enzymes to enhance fungal resistance has been widely practiced, e.g. introgression of rice chitinase cDNA into cucumber enhanced its resistance to *Botrytis cinerea* by suppressing the growth of fungi (Kishimoto *et al.* 2002). In *Brassica juncea*, *Rhizoctonia solani* infection induces the expression of BjCHI1, a chitinase enzyme. Transgenic *Solanum tuberosum* L. overexpressing either *BjCHI1* or *BjCHI1* and *HbGLU* (*Hevea brasiliensis* β-1,3-glucanase) exhibited significant inhibition of fungal growth (Chye *et al.* 2005). Chye *et al.* (2005) suggested that co-expression of proteins can effectively degrade fungal cell wall producing elicitors by initiating epidermal cell collapse and thus restricting further hyphal penetration. They also noted that there are small-sized proteins, e.g. plant defensins, that play an active role in plant defence against a variety of diseases. Of the various plant defensins, NaD1 from *Nicotiana alata* is a well-characterized antifungal protein and its overexpression increased the resistance of cotton to *Fusarium oxysporum* and *Verticillium dahlia*, resulting in an enhanced survival rate and yield (Gaspar *et al.* 2014).

Amongst the most commonly used herbicides are glyphosate and bromoxynil (3,5-dibromo-4-hydroxybenzonitrile). Glyphosate restricts growth by reducing aromatic amino acid biosynthesis while bromoxynil prevents photosynthesis by affecting PSII activity (Stalker *et al.* 1988). The development of glyphosate resistant crops would help plants resist the glyphosate and thus reduce yield losses. Research efforts have been successful in identifying and characterizing glyphosate resistance genes and to date various 5-enolpyruvylshikimate-3-phosphate (EPSP) resistant genes have been identified. Shah *et al.* (1986) developed glyphosate resistant petunia using the cauliflower mosaic virus 35S promoter, resulting in 20-fold amplification of the ESPS synthase gene. Similarly, Tian *et al.* (2013) developed a transgenic rice cultivar through the incorporation of *MdEPSPS*, a gene conferring glyphosate resistance, in *Malus domestica*, which they identified after five rounds of DNA shuffling and screening; amongst the eight mutations in the amino acid sequence of this gene only two were identified as site directed and important for glyphosate resistance. Stalker *et al.* (1988) isolated and cloned the *bxn* gene from the soil bacterium *Klebsiella ozaenae*. This gene codes for nitrilase and mediates conversion of bromoxynil to its primary metabolite form (3,5-dibromo-4-hydroxybenzoic acid), and when introduced into tobacco, enhanced bromoxynil resistance. Recently, Iwakami *et al.* (2014) isolated two cytochrome P450 genes of CYP81A, i.e. *CYP81A12* and *CYP81A21*, from a noxious weed *Echinochloa phyllopogon* that is resistant to the herbicides bensulphuron-methyl and penoxsulam, and developed transgenic *Arabidopsis* expressing either of these genes that showed enhanced herbicide resistance through the *O*-demethylation of herbicides. These results indicate that the characterization and understanding of molecular mechanisms and the development of resistant crops can help withstand the devastating effects of pathogens and pests and provide an important alternative to chemical dependent agriculture.

### Nutrient stress

Changes in environmental conditions have a direct influence on nutrient uptake and assimilation in plants (Lopez-Arredondo *et al.* 2013). Amongst the various nutrient deficiencies, commonly reported deficiencies include those of iron, zinc and calcium, while other mineral deficiency disorders are believed to be rare (Taiz and Zeiger 2010). Most chemical fertilizers, which are enriched with desired nutrients, may improve biomass, but their role in improving the nutritional value for consumption is minimized either through leaching, surface run-off, volatilization or microbial consumption. Increasing nutrient use efficiency (NUE) amongst crops through efficient means is crucial to prevent mineral losses. Extensive contributions from conventional breeding with regard to improving NUE in crops have been made during the past few decades (Ashraf *et al.* 2011), but such achievements through advanced molecular techniques have not been numerous. Efficient working of transporters and enzymes involved in nutrient assimilation is essential for achieving enhanced nutrient uptake, and this has a direct influence on the crop yield status. For example, overexpression of glutamine synthetase gene (*GS1*) in wheat plants led to increased nitrogen accumulation in shoot and grains (Habash *et al.* 2001), whereas overexpression of *GS1-3* led to enhanced (30 %) kernel number in maize (Martin *et al.* 2006). At the molecular level, there are very few reports in the literature pertaining to the mechanisms and associated genes involved in nutrient transport and assimilation. However, it is widely accepted that TFs and associated kinases are involved in these processes (Canales *et al.* 2014).

Efficient working of the ammonium transporter, OsAMT1, helps transgenic rice plants to achieve and maintain sufficient levels of ammonium, the major source of nitrogen for rice. This suggests the role of this transporter in enhancing NUE, growth and yield under optimal as well as suboptimal nitrogen conditions (Ranathunge *et al.* 2014). NRT1.1 functions as a nitrate sensor and can enhance high to low affinity nitrate transporters in the protein kinase CIPK23 dependent phosphorylation and dephosphorylation of intracellular threonine, thereby changing NRT1.1’s ability to mediate efficient nitrate transport (Parker and Newstead 2014). Moreover, nodule inception (NIN)-like protein (NLP) TFs are the master regulators of nitrate response, and upon binding with the nitrate responsive *cis*-element activate nitrate-responsive transcription, which is further modulated by nitrate signalling at the post-translational level. Suppression of NLP function results in impeded expression of several nitrate-inducible genes (Konishi and Yanagisawa 2013). Castaings *et al.* (2009) reported that *nlp7* mutants show impaired nitrate signal transduction, and that its expression pattern and function in sensing nitrogen are closely associated with each other. Kuo and Chiou (2011) suggested that micro-RNAs have a putative rolein regulating the nutrient starvation genes at post-transcriptional levels.

Nutrient rich cultivars can be selectively developed from the existing germplasm or through genetic manipulation. Tailoring of the genetic makeup of crops for improved nutrient levels is gaining interest as a means to reduce malnutrition. Microarray and sequence based transcription profiling technology to study gene expression changes in response to nutrient stress can yield meaningful results (Lee *et al.* 1999). Transient changes in gene expression in nutrient starved plants are well documented. Bi *et al.* (2007) reported the differential expression of genes under mild nitrogen stress that were acting as putative regulators of nitrogen stress responses in *Arabidopsis*. An *Arabidopsis* mutant defective in developing proper nitrogen stress responses showed altered transcriptional responses to nitrogen limitation because of the absence of a key regulatory gene, *NLA* (Peng *et al.* 2007). In rice and maize, a systems approach is being adopted, mainly aiming at profiling genes at transcriptional levels in response to individual or combined nutrient stress.

Genetic engineering approaches for enhancing NUE range from increasing the solubility of mineral nutrients and remobilization within the plant to transport and accumulation within storage organs. Recently, Zhou *et al.* (2014) developed transgenic soybean in which constitutive overexpression of *GmEXPB2* (β-expansin) increased leaf expansion and improved phosphate efficiency. Overexpression of expansin genes, e.g. *HvEXPB1* in barley (Kwasniewski and Szarejko 2006) and *OsEXPA17* in rice (Yu *et al.* 2011), improved phosphate uptake through induction of better root hair growth even under phosphate-deficient conditions. Remobilization of nutrients within a plant is critical for their survival, and manipulation of transporter genes for efficient remobilization of nutrients is an important strategy. For example, overexpression of the *GmPT1* transporter gene enhanced phosphate remobilization, yield and related attributes like phosphorus use efficiency and quantum yield in soybean (Song *et al.* 2014). To enhance NUE through genetic manipulations, a thorough understanding about the factors governing efficient mineral uptake and remobilization within the plant is necessary. The use of methods such as transcription profiling, analysis of mutants defective in their response to mineral deficiency and investigation of plants showing normal growth under nutrient stress are pre-requisites.

The biofortification of important seed crops for optimal accumulation of micronutrients has been the subject of intensive research for the last few decades (Akram *et al.* 2009; Akram and Ashraf 2011). For example, during a study on sunflowers, Akram *et al.* (2009) found that a foliage spray of potassium sulphate significantly improved shoot and leaf K^+^ contents, while no change was observed in leaf and root Mg^2+^, Ca^2+^ or N content under non-stress and saline conditions. Similarly, soil and foliar application of Zn and Fe at the rate of 4.0 and 2.0 mg kg^−1^, respectively, had a significant effect on plant available nutrients and nutrient concentration in wheat grain and straw (Naz *et al.* 2015).

The transgenic approach has been preferred over the conventional one for the biofortification of important crops, because it is a convenient and time and labour saving approach for overcoming nutrient deficiency problems (Zhu *et al.* 2007). Rice plants overexpressing the iron storage protein ferritin were reported to have increased seed iron content (Lucca *et al.* 2001). Adoption of GM crops is a time-consuming process as a number of policies and laws need to be adopted before the commercial release of such cultivars or varieties can occur. A cultivar or variety that is transgenic for one of the nutrients could involve up- or down-regulation of a cascade of genes involved therein, which could cause human health problems. A number of government agencies are working mostly in developed countries to review these issues. The transgenic approach is considered as an extremely useful tool in basic plant science research, but understanding of the gene networks and molecular physiology of plant responses to deficiency or excess of nutrients is a pre-requisite.

### Heavy metals

Heavy metal pollution and contamination is a major determinant of the global distribution of plant species as well as agricultural productivity, particularly in countries where the economy relies heavily on industry (Khan *et al.* 2014, 2015). Many plants show symptoms of heavy metal toxicity when a metal concentration surpasses a specific threshold level. In general, necrosis and stunted shoot growth are the first visible symptoms of heavy metal exposure (Liu *et al.* 2014).

Heavy metals interfere with the growth and physiology of plants in several ways. Heavy metals like lead (Pb) and cadmium (Cd) reduce the number of mitochondrial cristae leading to impaired oxidative phosphorylation. Upon binding to nucleic acids, heavy metals promote the aggregation and condensation of chromatin as well as impaired replication and transcription (Youssef and Azooz 2013). The affinity of Pb and Cd to sulphohydryl groups of enzymes leads to their inactivation. In addition, heavy metal stress leads to the enhanced production and accumulation of ROS and thus oxidative stress, which usually upsets the normal metabolism (Groppa *et al.* 2012). Phytoremediation is being extensively promoted as a means to remediate environmental contaminants such as heavy metals (Glick 2003). Moreover, rhizoremediation, which involves plants as well as their rhizospheric microbes, either naturally occurring or introduced, also helps to degrade or lower the levels of contaminants and promote normal plant growth (Gerhardt *et al.* 2009; Qadir *et al.* 2014).

Most heavy metal salts are hydrophilic and easily soluble in wastewater, so they are difficult to separate by physical separation methods. At low levels of heavy metals, physico-chemical methods can be ineffective or costly. Alternative methods include biosorption or bioaccumulation for the removal of heavy metals. The use of microorganisms and plants for remediation purposes is thus an effective strategy to overcome or minimize heavy metal pollution (Dixit *et al.* 2015). Despite the potential of these strategies to contribute to reclamation of contaminated soils, detailed information on the underlying mechanisms is not available in the literature and efforts to transform these strategies from successful laboratory or greenhouse trials to field natural sites are highly challenging. Two factors that make these strategies not very effective are (i) the multiple stress factors available in the field are not employed under laboratory and greenhouse studies and (ii) there is a lack of efficient and adequate methodologies and techniques that can be employed to ascertain whether or not the concentrations of different contaminants are decreasing (Gerhardt *et al.* 2009).

Phytoremediation is an ecofriendly, cost-effective and non-invasive strategy that is now being used extensively to clean up heavy metals from the environment or render them harmless. Various mechanisms, e.g. chelation, trafficking, compartmentation, etc. are employed for detoxification of toxic heavy metals and metalloids (Guo *et al.* 2008; Khan *et al.* 2014). Moreover, production of higher levels of high affinity ligands like phytochelatins (PCs) and metallothioneins (MTs), and cysteine rich thiol-reactive peptides, mediates detoxification by binding with toxic metals and metalloids (Gasic and Korban 2007; Guo *et al.* 2008; Pal and Rai 2010). Formation of PSc-metal or MT-metal complexes and their subsequent sequestration into the vacuole are essential for heavy metal tolerance. The enzyme phytochelatin synthase (PCS) mediates the synthesis of PCs using GSH or γ-glutamyl cysteine as a substrate (Cobbett and Goldsbrough 2002; Wunschmann *et al.* 2007). Effective research has been performed regarding genes encoding PCs, with several genes being cloned to date, e.g. *OsPCS1*, *TaPCS1*, *AtPCS1* and *CePCS1* from rice, wheat and *Arabidopsis* (Ha *et al.* 1999; Vatamaniuk *et al.* 1999; Gasic and Korban 2007), and *BjPCS1* and *AsPCS1* from the metal tolerant plants *B.juncea* and *Allium sativum*, respectively (Heiss *et al.* 2003; Zhang *et al.* 2005).

Guo *et al.* (2008) demonstrated that simultaneous overexpression of *AsPCS1* and *GSH1* (from *A. sativum* and *S. cerevisiae*) enhanced the tolerance of *A. thaliana* to heavy metals and metalloids. They further reported that single-gene transgenic lines showed higher tolerance and accumulated more Cd and As than wild type plants, while dual gene transgenic lines exhibited even more tolerance and accumulated (2-fold) more Cd and As compared with single gene transformants. The elevated production of GSH and PCs resulted in accumulation and tolerance to Cd and As (Li *et al.* 2004). Plants used in phytoextraction should have an inherent capacity to accumulate and tolerate high contents of metalloids in aboveground biomass (Pajevic *et al.* 2016). In addition, they should have fast growing adaptability and biomass, and ideally be repulsive towards herbivores so that transmission of toxic metals to various components of the food chain can be avoided. Besides these basic characteristics, hyper-accumulator plants have should have a profusely branched root system, wide geographical distribution and be easy to cultivate as well as harvest.

Genetic manipulations for developing specific morphological characteristics supported by unique anatomical efficiencies for the accumulation of metalloids are being intensively studied (Kotrba *et al.* 2009). Tolerant plant species show increased uptake and metal binding capacity at intracellular sites, and efficient sequestration into the vacuole for deposition and detoxification that is controlled in a highly regulated manner by a set of gene products (Peng *et al.* 2014). For instance, yeast protein YCF1 mediates the sequestration of Pb and Cd into the vacuole. Transgenic *A. thaliana* plants overexpressing YCF1 were found to be tolerant to Pb and Cd (Song *et al.* 2003). In addition, enhanced translocation of metals to aboveground plant parts via the apoplast or symplast, and their subsequent extrusion to metabolically less active tissues like trichomes, was also found to contribute to enhanced metal tolerance as well as remediation (Clemens *et al.* 2002).

The role of PCs and MTs in heavy metal detoxification has been well documented. Gonzalez-Mendoza *et al.* (2007) reported that increased expression of *AvPCS* and *AvMt2* in *Avicennia germinans* under Cd and Cu stress indicates that the PCs and MTs are involved in a coordinated detoxification response mechanism employed for removal of non-essential metals. Scientists are continuously striding towards enhancing the detoxifying potential of crop plants through manipulating the genes coding for PCs and MTs, e.g. *Nicotiana tabacum* (Sylwia *et al.* 2010) and *B.juncea* (Gasic and Korban 2007) overexpressing *AtPCS1* (PCS) showed improved tolerance to Cd by maintaining higher levels of PCs in the cytosol and vacuole. *Arabidopsis thaliana* overexpressing *PCs1* showed higher resistance to Cd and arsenic (As) and accumulated more biomass (Verbruggen *et al.* 2009). Moreover, concentrations of Cd and As decreased while that of thiol peptide increased in shoot biomass (Li *et al.* 2004). Besides increasing tolerance, transgenic plants accumulated less metal content in the aboveground biomass (Gasic and Korban 2007).

Overexpression of *AtPCS1* in *N.tabacum* harbouring the *Agrobacterium rhizogenes rolB* oncogene enhanced its tolerance to Cd; tolerance was further enhanced when the culture was supplemented with GSH (Pomponi *et al.* 2006). Transgenic plants showing increased expression of *O*-acetylserine (thiol) lyase (OASTL), a key enzyme that catalyzes cysteine formation (from sulphide and O-acetylserine) and a key limiting step in the production of GSH, showed high tolerance to heavy metals (Ning *et al.* 2010). *Nicotiana tabacum* plants expressing the wheat (*Triticum aestivum*) *OASTL* gene, cys1, and exposed to SO_2_ maintained high levels of Cys and GSH as well as higher rates of accumulation of Cu/Zn SOD transcripts.

The advancement in transgenic approaches (individual or combination) could be a successful means to promote phytoextraction of toxic metalloids (Se and As) and metals, particularly Cu, Pb and Cd in the aboveground plant organs and to promote tissue up-take involving metal transporters, high production of enzymes and the production of metal-detoxifying chelators including PCs and MTs. Advances in the mechanistic basis of a transgenic approach would help provide a better understanding of the genetic basis of resistance or tolerance and hyperaccumulation of metals and metalloids, means of translocation and other environmental factors influencing phytoremediation, because these all hinder its implementation.

## Conclusions and Future Prospects

Several intrinsic protective mechanisms are triggered in plants when they are exposed to various environmental stresses (Sadiq *et al.* 2017). Deciphering the regulatory mechanisms involved in initiating these tolerance pathways has remained under intensive research for decades. Testing of the validity of these assumptions has provided new insights towards the better understanding and elucidation of stress-induced changes in plants. Plant physiologists and biochemists have remained the key players in elucidating the basics of these mechanisms, which are now being extensively explored at the genetic and molecular levels using various molecular, genomic and biotechnological approaches.

The identification and selection of key stress responsive genes and their subsequent introgression for developing resistant crop cultivars through conventional breeding protocols are time-consuming. Plant biotechnology, despite being costly in comparison with conventional breeding, is very efficient. Several stress responsive genes have been identified and successfully introduced into other crops to create transgenic crops with enhanced stress tolerance. However, it is important to point out here that during the development of a transgenic crop variety, care is taken to introduce genes that result in enhanced tolerance to multiple stresses, specifically at the whole plant level. This requires the development of sets of markers designed to enhance stress tolerance.

The advantages of biotechnology in the development of transgenic plants for efficient crop varieties are undoubtedly enormous, but their commercialization after proper field testing is still an unavoidable reality. In addition, risk assessment of transgenic plants/crops is one of the preliminary steps required before the release or use of transgenic plants. The set standard all over the world explains the risk and official registration of plants and plant products has to be under taken. In addition, the risks to the environment from the transgenic crop plants must be examined with many field tests prior to commercialization, with institutional assessments, decisions on plants or varieties and adequate management practices in place to tackle inherent risks. For decision making, risk assessment must be followed in a scientific, sound and transparent manner. There are many operational governmental regulations in many countries for the safety assessment of GM crops. Furthermore, there are some international agreements that regulate the cultivation and commercialization of transgenic plants and their derivatives. All over the world, the major objective of these regulations and risk assessment strategies is focused on protecting the environment and human/animal health. The adoption of transgenic plants entirely depends on the assessments of the risks or benefits, regulatory approval, cost and time period, commercialization as well as the economic status, requirements and values of different countries.

## Sources of Funding

Not applicable. 

## Contributions by the Authors

P.A. conceived the idea, N.A.A., M.A. and L.W. compiled the data, M.A.A. and M.N.A. led the writing of the manuscript with inputs from all co-authors.

## Conflict of Interest Statement

None declared.
